# Co-Expression of miR155 or LSD1 shRNA Increases the Anti-Tumor Functions of CD19 CAR-T Cells

**DOI:** 10.3389/fimmu.2021.811364

**Published:** 2022-01-03

**Authors:** Jing Zhang, Jingjing Zhu, Genhui Zheng, Qianyu Wang, Xiaorui Li, Yaru Feng, Fengqin Shang, Siqi He, Qiyao Jiang, Bingjie Shi, Dong Wang, Zhiwei Cao, Jianxun Wang

**Affiliations:** ^1^ School of Life Sciences, Beijing University of Chinese Medicine, Beijing, China; ^2^ School of Life Sciences and Technology, Tongji University, Shanghai, China; ^3^ School of Life Sciences, Tsinghua University, Beijing, China; ^4^ School of Basic Medical Sciences, Chengdu University of Traditional Chinese Medicine, Chengdu, China; ^5^ Shenzhen Research Institute, Beijing University of Chinese Medicine, Shenzhen, China

**Keywords:** CAR-T cells, CD19, retroviral vector, miR155, LSD1 shRNA

## Abstract

Chimeric antigen receptor (CAR) T cells targeting CD19 antigen have produced remarkable clinical outcomes for cancer patients. However, identifying measures to enhance effector function remains one of the most challenging issues in CD19-targeted immunotherapy. Here, we report a novel approach in which a microRNA (miRNA) or short-hairpin RNA (shRNA) cassette was integrated into CAR-expressing retroviral vectors. Using this system, we generated anti-CD19 CAR-T cells co-expressing miR155 or LSD1 shRNA and found that anti-CD19 CAR-T cells with miR155 upregulation or LSD1 downregulation exhibited increased anti-tumor functions *in vitro* and *in vivo*. Transcriptional profiling analysis by RNA sequencing revealed the targets of miR155 and LSD1 in anti-CD19 CAR-T cells. Our experiments indicated that introduction of miRNA or shRNA expression into anti-CD19 CAR T-cells might be an effective strategy to improve the anti-tumor effects of CAR-T cell therapy.

**Graphical Abstract:**
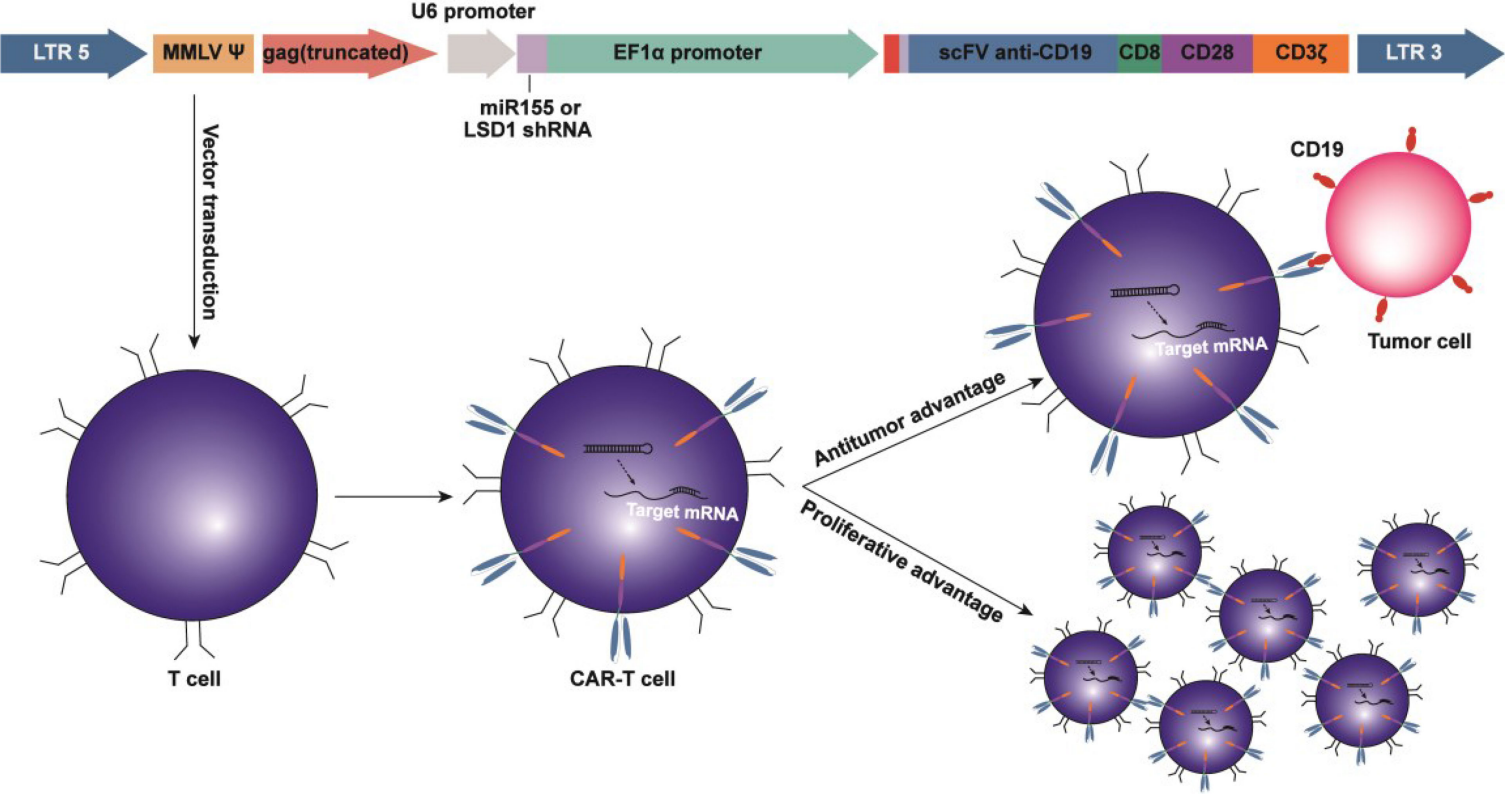


## Introduction

CD19-specific chimeric antigen receptor (CAR)-T cell therapy has achieved impressive progress in the treatment of hematopoietic malignancies, especially relapsed/refractory diffuse large B-cell lymphoma and relapsed/refractory B acute lymphoblastic leukemia since the US Food and Drug Administration approved the first two CD19 CAR-T cell treatments (axicabtagene ciloleucel and tisagenlecleucel) (1–3). However, some lymphoma patients show recurrence after CAR-T cell therapy (4). Therefore, further improvement in the efficacy of CAR-T cells for lymphoma treatment is required.

One of the major obstacles of CAR-T cell therapy is the limited expansion and persistence of CAR-T cells, which potentially hinder the long-term anti-tumor response in patients (5). Many strategies have been developed to improve the anti-tumor effects of CAR-T cells by extending the lifespan of CAR-T cells through regulation of CAR-T cell differentiation and inhibition of CAR-T cell senescence (6). The important role of epigenetics in the regulation of T cell function and activity has been verified (7, 8).

Mammalian microRNAs (miRs) are small (~22 nucleotides) noncoding RNA oligonucleotides that regulate gene expression at the posttranscriptional level by targeting the corresponding mRNAs (9). miRNAs have been demonstrated to exhibit critical functions in multiple biological processes. For example, miR155 plays a critical role in T cell activation, lymphocyte homeostasis and immune responses (10). miR155 is required for optimal effector CD8+ T cell accumulation, memory cell differentiation and anti-tumor activity (11, 12), as well as CD4+ T cell functions, including activation, function, apoptosis and differentiation (13). miR155 regulates multiple transcription factors that are involved in these processes, such as Fosl2, SHIP1 and SOCS1 (14). Enhanced miR155 expression was demonstrated to promote the long-term persistence and expansion of T cells during chronic infection (15). In addition, miR155 overexpression was shown to modulate T cell fitness and metabolism with improved anti-tumor activity (16, 17). ﻿Therefore, introduction of miR155 may represent a potential strategy to improve the anti-tumor activity of CAR-T cells.

Lysine-specific demethylase 1 (LSD1, also known as KDM1A, AOF2 and BHC110) is a histone lysine-specific demethylase that removes mono- or dimethylation of H3K4 or H3K9 in the presence of flavine-adenine dinucleotide. LSD1 plays an important role in tumorigenesis and is a potential target in anti-tumor immunity (18). LSD1 inhibition improved tumor immunogenicity and promoted T cell infiltration, and thus inhibition of LSD1 in combination with PD-1/PD-L1 blockade was suggested as a novel cancer treatment strategy (19). Numerous LSD1 inhibitors are currently undergoing clinical assessment for cancer therapy (20). However, research on the T cell response after LSD1 inhibition in cancer therapy is limited.

MiR155 and LSD1 were important epigenetic regulators with anti-tumor functions. MiR155 targets and negatively regulates LSD1 in both HFLS and MH7A cells, and upregulation of miR155 decreases the expression of LSD1 in rheumatoid arthritis (21). This is consistent with our previous study (22), indicating that LSD1 may be the direct target of miR155, suggesting that effects of over-expression of miR155 is at least partially by LSD1 down-regulation.

In this study, we engineered CAR-T cells expressing either miR155 or short-hairpin RNA (shRNA) targeting LSD1 and explored the effects of miR155 expression or LSD1 knockdown on the efficacy of CAR-T cell therapy. Our results revealed that miR155 upregulation or LSD1 downregulation enhanced the anti-tumor functions of CD19-specific CAR-T cells. Our approach takes advantage of epigenetic regulators to modulate CAR-T cell activity and has significant implications for cancer immunotherapy.

## Materials and Methods

### Cell Culture

Phoenix-ECO cells and PG13 cells were obtained from the American Type Culture Collection (ATCC) and cultured in Dulbecco’s Modified Eagle Medium (DMEM, Gibco, USA) with 10% FBS. The Raji cell line, which are lymphoblast-like cells derived from human Burkitt’s lymphoma, was obtained from ATCC. Raji cells stably expressing firefly luciferase (Raji-luc) were obtained from Beijing Vitalstar Biotechnology Company. Both cell lines were cultured in RPMI 1640 medium (Gibco) with 10% FBS. hCD19-SW620 cells expressing the human CD19 antigen were obtained from Tsinghua University (Beijing, China) and cultured in DMEM with 10% FBS.

### Retroviral Vector Construction, Packaging and Titer Measurement

Anti-CD19 CAR molecule consisted of anti-CD19 single-chain variable fragments (scFv) with variable heavy and light chains (FMC63) separated by a (G4S)3 linker, followed by a CD8 hinge and transmembrane domain and CD28 and CD3ζ intracellular signaling domains. The construct contained a signal peptide and myc tag upstream of the anti-CD19 CAR sequence. The myc tag was used to detect the transduction efficiency.

We generated three constructs with MFG retroviral vector: a construct expressing U6 RNA and the anti-CD19 CAR sequence, a construct expressing miR155 and the anti-CD19 CAR sequence and a construct expressing LSD1 shRNA and the anti-CD19 CAR sequence. The miR155 sequence was 5’-CTGTTAATGCTAATCGTGATAGGGGTTCTTGCCTCCAACTGACTCCTACATATTAGCATTAACAGTTTTT-3’ (MI0000681, miRBase, http://www.mirbase.org/cgi-bin/mirna_entry.pl?acc=MI0000681), the LSD1 shRNA-1 sequence was 5’-CCGGGCCTAGACATTAAACTGAATACTCGAGTATTCAGTTTAATGTCTAGGCTTTTTG-3’ (TRCN0000046068, GPP Web Portal, https://portals.broadinstitute.org/gpp/public/gene/search), and the LSD1 shRNA-2 sequence was 5’- CCGGGCTCCAATACTGTTGGCACTACTCGAGTAGTGCCAACAGTATTGGAGCTTTTTG-3’ (TRCN0000046069, GPP Web Portal, https://portals.broadinstitute.org/gpp/public/gene/search).

For retroviral vector packaging, a two-step method involving the sequential use of Phoenix-ECO cells and PG13 cells was used to produce stable PG13 retroviral vector producer cell lines. Retroviral vectors were harvested and used for the transduction of activated human T cells.

Vector titers were determined by two-step quantitative reverse transcription-polymerase chain reaction (RT-qPCR) analysis. First, RNA was purified from cell supernatants (QIAamp^®^ Viral RNA Mini Kit, QIAGEN, Germany), followed by cDNA synthesis (QuantiNova Reverse Transcription Kit, QIAGEN). RT-qPCR analysis was performed using the QuantStudio6 Flex system (Applied Biosystems, Foster City, CA, USA) and SYBR Green Mix (QuantiNova SYBR^®^ Green PCR Kit, QIAGEN). The primer sequences were provided in **Supplementary Table 1**.

### T Cell Isolation, Activation and Transduction

Human peripheral blood mononuclear cells were isolated and purified from peripheral blood of healthy human donors using Lymphoprep (Stemcell, Canada). Cells were then activated with 100 ng/mL human anti-CD3 (clone OKT3; Sino Biological, Beijing, China) and 100 U/mL IL-2 (Sino Biological) in AIM-V^®^ + AlbuMAX^®^ (bovine serum albumin) Serum-free Medium (Gibco) supplemented with 10% fetal bovine serum (FBS, Gibco) for 48 hours at 37°C in a 5% CO_2_ incubator. T cells were expanded for 10-20 days at a concentration of 1×10^6^ cells/mL in the presence of continuous IL-2 stimulation.

After activation for 48 hours, T cells were transduced with CAR retroviral vector and cultured in a 5% CO_2_ incubator at 37°C for 48 hours. The transduction efficiency was detected by flow cytometry analysis (CytoFLEX, Beckman Coulter, USA). The retroviral vector copy number integration per T cell was detected by qPCR; the primer sequences were provided in **Supplementary Table 1**.

### miR155 and LSD1 mRNA Expression Analysis

For the detection of miR155 expression, miRs were isolated from cells using the miRcute miRNA Kit (TIANGEN, Beijing, China). Reverse transcription was performed using the TaqMan MicroRNA Reverse Transcription Kit (Applied Biosystems, USA) and specific primers for miR155 and U6 RNA provided by Applied Biosystems. The expression of miRNAs was measured by RT-qPCR using the TaqMan Universal Master Mix II with UNG (Applied Biosystems, USA).

For the detection of LSD1 mRNA expression, total RNA was isolated from cells using the RNeasy Mini Kit (QIAGEN). cDNA was then synthesized from RNA (QuantiNova Reverse Transcription Kit, QIAGEN) and subjected to RT-qPCR using SYBR qPCR mix (QuantiNova SYBR^®^ Green PCR Kit, QIAGEN). The primer sequences are provided in **Supplementary Table 2**.

RT-qPCR reactions were performed in triplicate using the QuantStudio6 Flex system (Applied Biosystems). The miR155 expression levels were normalized to U6 small nucleolar RNA levels, and the LSD1 mRNA expression levels were normalized to *ACTB* mRNA levels. Fold changes in expression were calculated with the 2-ΔΔCT method.

### Tumor Cell Co-Culture and Measurement of Interferon-γ (IFN-γ), Tumor Necrosis Factoralpha (TNF-α) and Interleukin (IL)-2

A total of 80,000 CAR-T cells (effector, E) and tumor cells (target, T) were co-cultured at a 1:1 effector/target (E/T) ratio for 12 hours in a 96-well plate (0.2 mL/well). Cell culture supernatants were analyzed by enzyme-linked immunosorbent assays with a Human IFN-γ ELISA Kit, Human TNF-α ELISA Kit and Human IL-2 ELISA Kit (all Proteintech, Wuhan, China). Samples were measured on a SpectraMax Series Multi-Mode Microplate Reader (model i3x, Molecular Devices, CA, USA) at a wavelength of 450 nm with a correction wavelength of 630 nm. Data were analyzed using SoftMax Pro 6.4.2 software (Molecular Devices).

### CAR-T Cell Proliferation, CD4+/CD8+ T Cell Detection and Memory T Cell Differentiation Detection *In Vitro*

CAR-T cells were pre-labeled with the intracellular fluorescent label carboxyfluorescein diacetate succinimidyl ester. Covalently bound carboxyfluorescein diacetate succinimidyl ester is divided equally between daughter cells, enabling the discrimination of successive cell division rounds. After culturing labeled CAR-T cells with Raji cells at an E/T ratio of 1:2 for 24 hours, samples were analyzed by flow cytometry analysis by staining with APC labeled anti-human CD3 antibody (clone HIT3a, BioLegend). The proliferation assays and carboxyfluorescein diacetate succinimidyl ester-staining were performed in the continuous presence of IL-2.

FITC-labeled anti-human CD4 antibody (clone OKT4, BioLegend) and PerCP-Cyanine5.5-labeled anti-human CD8 antibody (clone SK1, BioLegend) were used to detect CD4+/CD8+ T cells by FLOW CYTOMETRY ANALYSIS.

PE/Cyanine7-labeled anti-human CD45RO antibody (UCHL1, BioLegend) and PE-labeled CD62L antibody (DREG-56, BioLegend) were used to differentiate memory and effector T cell populations; central memory T (T_CM_) cells were defined as cells expressing CD45RO and CD62L.

Flow cytometry analysis was conducted on a CytoFLEX Cell Analyzer (Beckman Coulter), and data were analyzed using CytoFLEX Software (Beckman Coulter).

### Cytotoxicity Assay

For the apoptosis assay, CAR-T cells or non-transduced T cells (PanT) were co-cultured with Raji cells (4×10^4^) at E/T ratios of 1:1, 1:2, 1:4, 1:8 and 1:16 for 12 hours in a 96-well plate (0.2 mL/well), and then cells were stained with APC-labeled anti-human CD3 antibody (clone HIT3a, BioLegend) and FITC-labeled Annexin V (Gene-Protein Link, China) for 30 minutes. Data were collected on a CytoFLEX Cell Analyzer (Beckman Coulter) and analyzed with CytoFLEX Software (Beckman Coulter). The cytolytic activity of CAR-T cells was calculated as follows: percent cytolysis = (percentage of CD3^-^/Annexin V^+^ co-cultured cells) − (percentage of Annexin V^+^ monocultured tumor cells).

For the stress test, repetitive antigen stimulation *in vitro* was performed. Raji and CAR-T cells were co-cultured at an E/T ratio of 1:1 in the presence of IL-2 (100 U/mL). After 48 hours, some CAR-T cells were harvested for the apoptosis assay and analyzed by flow cytometry analysis as described above. The remaining CAR-T cells were then transferred to a fresh culture of Raji cells. After each round, the apoptosis assay was performed, and three rounds of transfer over 6 days were conducted.

For the luciferase assay, Raji-luc cells (4×10^4^) were co-cultured with CAR-T cells or PanT cells at E/T ratios of 1:1, 1:2, 1:4, 1:8 or 1:16 for 12 hours in a 96-well white assay plate (Corning, NY, USA) (0.1 mL/well). Next, 100 µl of Luciferase Assay Reagent (Promega, USA) were added, and cells were incubated at room temperature for 5 minutes. The fluorescence was measured on a SpectraMax Series Multi-Mode Microplate Reader (model i3x; Molecular Devices), and data were analyzed using SoftMax Pro 6.4.2 software (Molecular Devices). Lysis was calculated according to the following formula: % lysis = (experimental lysis − spontaneous lysis) × 100/(maximum lysis − spontaneous lysis).

For the real-time cytotoxicity assay, 50 μl of AIM-V medium were added to each well of a 96-well E-plate (Acea Biosciences, San Diego, CA, USA) for the baseline measurement. Adherent target SW620 cells were then seeded at a density of 2×10^4^ cells/50 μl medium and monitored in culture for 48 hours with the impedance-based real-time cell analysis (RTCA) xCELLigence system (Acea Biosciences). After 48 hours, CAR-T or PanT cells (5×10^3^ cells in 50 μl of medium) were added to each well. The cells in the E-plates were monitored with the RTCA system over time.

### Xenograft Models

Severe combined immune-deficient NPG/Vst mice (members of the NOD-Prkdc^scid^ Il2rg^null^ family, obtained from Beijing Vitalstar Biotechnology) were used for the mouse xenograft model. NPG/Vst female mice (6-8 weeks old) were inoculated intravenously (*i.v.*) with 1×10^6^ Raji-luc cells. After 4 days, the mice were randomized to seven groups of six mice each: control, PanT, anti-CD19 CAR-T, RNAU6 anti-CD19 CAR-T, miR155 anti-CD19 CAR-T, LSD1 shRNA-1 anti-CD19 CAR-T, and LSD1 shRNA-2 anti-CD19 CAR-T. Animals then received a dose of 1×10^8^/kg CAR-T cells or PanT cells by *i.v.* injection (that was about 2×10^6^ CAR-T cells or PanT cells per mice), followed by a second dose at 7 days after the first injection. And the experiments were terminated at the 52nd day.

Tumors were monitored twice a week by *in vivo* bioluminescence imaging of ventral views (multi-functional *in vivo* imager, Molecular Devices, USA). Before imaging, the mice were administered 150 mg/kg VivoGlo Luciferin *In Vivo* Grade (Promega) suspended in 200 μl PBS by intraperitoneal injection. After 3 minutes, the mice were placed in the anesthesia box of the Matrx Animal Anesthesia Ventilator System (VMR, Matrx, USA) with 1.5% isoflurane for 3 minutes. Bioluminescent imaging acquisition was then performed at the medium binning level with a 5 minutes exposure time. MetaMorph software (Molecular Devices) was used to acquire and quantify the bioluminescence imaging datasets. Tumor bulk was measured by MetaMorph imaging.

For the measurement of serum cytokine concentration, blood samples were taken from the tail veins of NPG mice on the 7th day after the second CAR-T cell injection. Serum IFN-γ concentration was measured using the Human IFN-γ ELISA Kit (Proteintech).

For the assessment of T cell persistence, blood samples were taken from the tail veins of NPG mice at multiple time points during the experiment, including the 52nd day. CD3+T cells were detected by flow cytometry analysis using Brilliant Violet 785™-labeled anti-human CD3 antibody (clone YCHT1, BioLegend).

The animal experiment protocol was approved by the Biomedical Research Ethics Committee of the Beijing University of Chinese Medicine (No. BUCM-4-2021010703-1004). Animal care and experiments strictly adhered to the American Physiological Society’s Guiding Principles for the Care and Use of Vertebrate Animals in Research and Training.

### RNA Sequencing (RNA-Seq) and Analysis

Total RNA was isolated from CAR-T cells using TRIZOL reagent (Invitrogen, USA) following standard protocols. The RNA-seq and analysis was performed by CapitalBio Technology Inc (Beijing, China). Predicted miR155 targets were acquired using the TargetScan database (http://www.targetscan.org/). The protein-protein interaction was analyzed by STRING (https://string-db.org/cgi/input.pl). The RNA sequencing data were deposited in NCBI Trace Archive NCBI Sequence Read Archive (https://www.ncbi.nlm.nih.gov/biosample/22548796, https://www.ncbi.nlm.nih.gov/biosample/22548797, https://www.ncbi.nlm.nih.gov/biosample/22548798, https://www.ncbi.nlm.nih.gov/biosample/22548799, https://www.ncbi.nlm.nih.gov/biosample/22548800, https://www.ncbi.nlm.nih.gov/biosample/22548801, https://www.ncbi.nlm.nih.gov/biosample/22548802, https://www.ncbi.nlm.nih.gov/biosample/22548803, https://www.ncbi.nlm.nih.gov/biosample/22548804, https://www.ncbi.nlm.nih.gov/biosample/22548805, https://www.ncbi.nlm.nih.gov/biosample/22548806, https://www.ncbi.nlm.nih.gov/biosample/22548807).

### Statistical Analysis

Statistical analyses were performed using GraphPad Prism7 software. Data were shown as means ± standard deviation. The unpaired Student’s t-test was used to determine statistically significant differences between two groups. All experiments were performed at least three times to establish reproducibility using at least independent donors. *p* < 0.05 was considered statistically significant.

## Results

### Co-Expression of miR155 or LSD1 shRNA With Anti-CD19 CAR in Human T Cells

**Figure 1:**
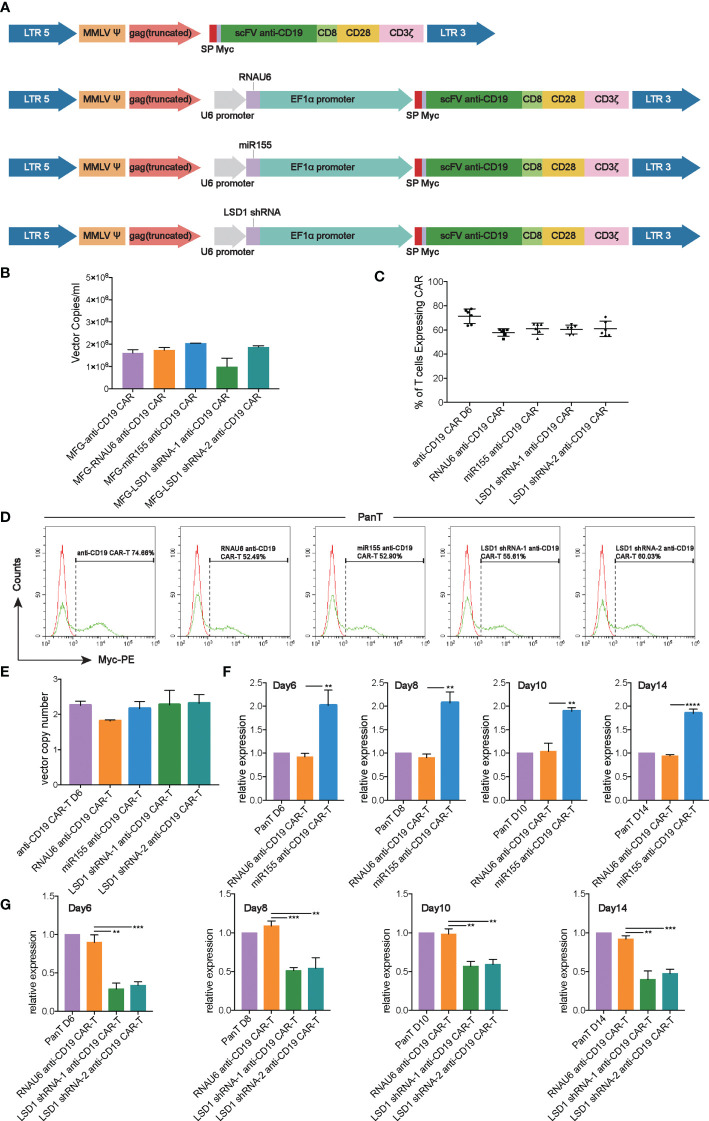
Construction and expression of anti-CD19 CAR. **(A)** The construction of anti-CD19 CAR. **(B)** The retroviral vector copies detected by RT-qPCR (n=3 donors). **(C)** Transduction efficiency of human T cells with anti-CD19 CAR retroviral vectors measured by flow cytometry analysis (n=6 donors). **(D)** Representative flow cytometry analysis profiles of transduction efficiency in an independent donor out of 6 donors. **(E)** The vector copy number integration per T cell determined by RT-qPCR (n=3 donors). **(F)** The expression of miR155 detected by TaqMan^®^ Assay-based RT-qPCR at the 6th, 8th,10th, 14th day after T cell isolation (n=3 donors). **(G)** The expression of LSD1 detected by SYBR Green-based RT-qPCR at the 6th, 8th,10th, 14th day after T cell isolation (n=3 donors). **(F, G)** ﻿Values were expressed as the means ± SD. Unpaired t test was performed. **p<0.01; *p<0.05; compared to RNAU6 anti-CD19 CAR-T cells.

We developed a strategy to generate CAR-T cells that simultaneously expressed anti-CD19 CAR with either miR155 or LSD1 shRNA. Two shRNA sequences against different sites of LSD1 mRNA were designed. The vector consisted of an U6 promoter followed by the sequence for miR155 or LSD1 shRNA and an EF1α promoter followed by a second-generation anti-CD19 CAR sequence encoding anti-CD19 scFv (FMC63), a CD8 hinge and transmembrane domain, a CD28 co-stimulation domain and a CD3ζ activation domain (**Figure 1A**). We used a PG13-based packaging cell line for the stable production of retroviral vectors, and retroviral vector producer clones were established. No statistically significant differences in vector copies between the groups were observed (**Figure 1B**).

After activation with immobilized anti-CD3 antibody, highly purified human T cells (**Figure S1A**) were transduced with the retroviral vectors, and the expression of anti-CD19 CAR was detected. The results indicated a high transduction efficiency of the anti-CD19 CAR retroviral vectors, and the expression of miR155 or LSD1 shRNA had no effect on the expression of anti-CD19 CAR (**Figures 1C, D**). Additionally, all CARs exhibited the proper vector copy number integration, which was possibly associated with a decreased risk of retroviral vector insertional mutagenesis (23) (**Figure 1E**). There were no significant differences in the ratios of CD4^+^/CD8^+^ T cells between the groups whether cultured alone or cultured with Raji cells (**Figure S1B**). The over-expression of miR155 and the downregulation of LSD1 mRNA by shRNA were confirmed by RT-qPCR. The results showed the predicted overexpression of miR155 and the downregulation of LSD1 mRNA in human T cells along the experimental timeline (**Figures 1F, G**).

### Anti-CD19 CAR-T Cell Cytolytic Activity Against Human CD19+ Cells *In Vitro*

We next evaluated the cytolytic activity of anti-CD19 CAR-T cells to verify the evaluation method and show how cytotoxic assays work. Raji cells expressing human CD19 (**Figure S1C**) were co-cultured with anti-CD19 CAR-T cells. Flow cytometry analysis results demonstrated that anti-CD19 CAR-T cells exhibited a higher cytolytic activity compared with un-transduced (PanT) cells with an increase in E/T ratio (**Figure S1D**). We further evaluated the cytotoxicity of anti-CD19 CAR-T cells against luciferase-expressing Raji cells (Raji-luc cells) (**Figure S1C**) by flow cytometry analysis and luciferase assay, and the results showed a similar cytolytic activity (**Figure S1E**). In additional experiments, adherent hCD19-SW620 cells with ectopic human CD19 expression were used as target cells for anti-CD19 CAR-T cells and analyzed by RTCA (**Figure S1C**). The results showed that hCD19-SW620 cells were efficiently killed by anti-CD19 CAR-T cells (**Figure S1F**). Furthermore, IFN-γ release of anti-CD19 CAR-T cells was significantly enhanced compared to PanT cells after co-culture with tumor cells at a 1:1 effector/target (E/T) ratio for 12 hours (**Figure S1G**). Consistent with the cytokine response, there was an increase in anti-CD19 CAR-T cell expansion and survival rates as measured by cell counts every 48 hours (**Figure S1H**).

### miR155 Overexpression Enhances the Function of CD19-Specific CAR-T Cells *In Vitro*

**Figure 2:**
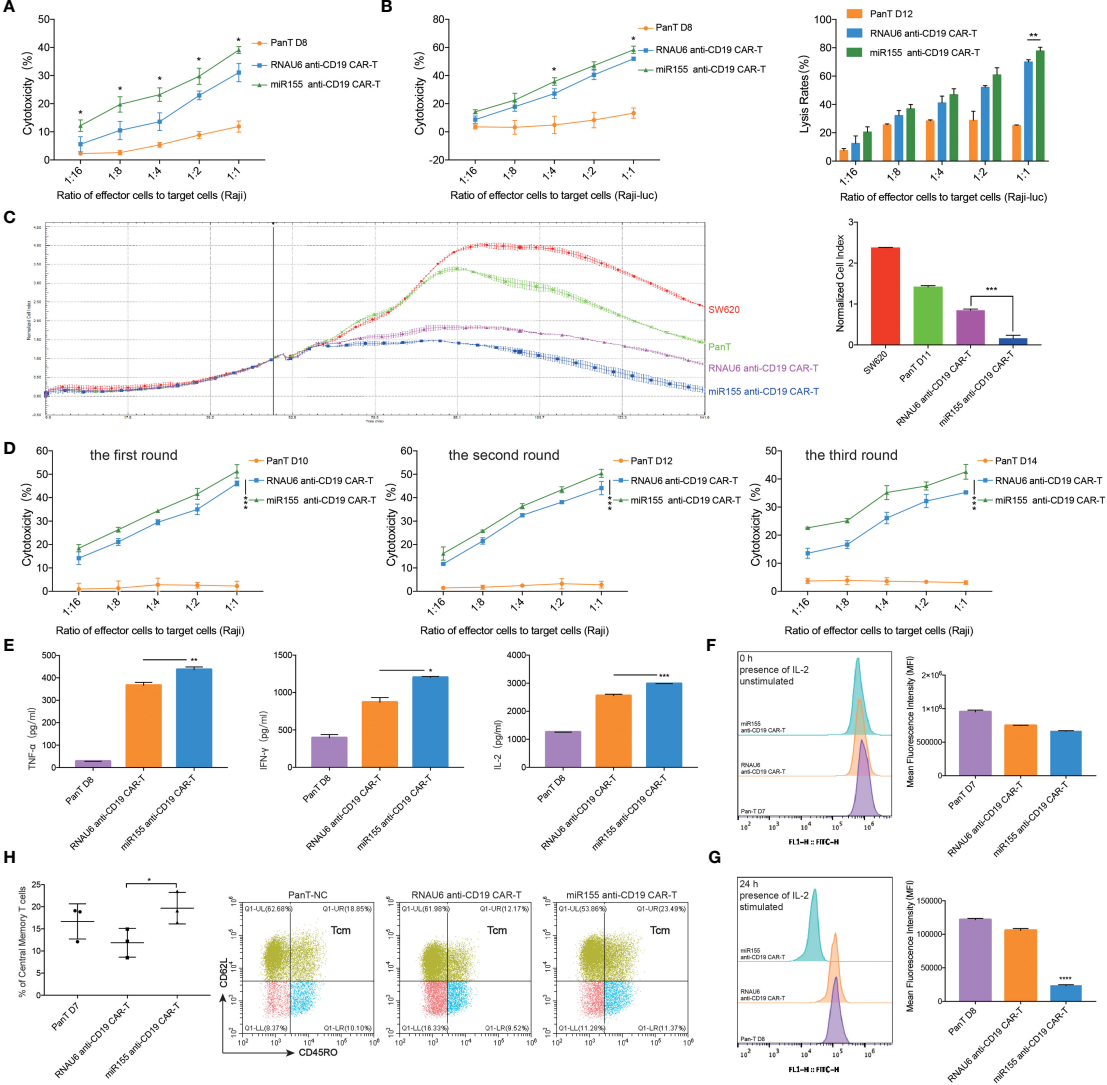
miR155 overexpression enhanced the function of anti-CD19 CAR-T cells *in vitro*. **(A)** Apoptosis assay. miR155 co-expressed anti-CD19 CAR-T cells were co-cultured with Raji cells at a gradient of E/T ratio for 12 hours. The apoptosis signals (Annexin V) of target cells were measured by flow cytometry analysis (n=3 donors). **(B)** The antitumor activity of miR155 co-expressed anti-CD19 CAR-T cells against Raji-luc cells detected by apoptosis assay (left) and luciferase assay(right) (n=3 donors). **(C)** The antitumor activity of miR155 co-expressed anti-CD19 CAR-T cells against hCD19-SW620 cells detected by RTCA. The RTCA system was used to monitor CAR-T cells and hCD19-SW620 cells at an E/T ratio of 1:4 in the E-plates with impedance plotted over time (left). The quantitation of normalized cell index at 141 hours was presented (right) (n=3 donors). **(D)** The stress test with repetitive antigen stimulation *in vitro*. CAR-T cells and Raji cells were co-cultured at an E/T ratio of 1:1 for 48 hours with no CD19^+^ cells detected. Then, part of CAR-T cells were seeded for the apoptosis assay. The rest of CAR-T cells were then moved to a fresh set of Raji cells at a 1:1 E/T ratio. After each round, the apoptosis assay was proceeded, and 3 rounds of transfer over 6 days were performed (n=3 donors). **(E)** The secretory level of TNF-α (left), IFN-γ (middle) and IL-2 (right) in CAR-T cells after the co-culture with Raji cells at an E/T ratio of 1:1 for 12 hours, detected by ELISA (n=3 donors). **(F)** The proliferation of miR155 co-expressed anti-CD19 CAR-T cells measured by carboxyfluorescein diacetate succinimidyl ester method. The stimulation referred to co-culturing with Raji cells at an E/T ratio of 1:2. The assay included continuous presence of IL-2. The 0 hour carboxyfluorescein diacetate succinimidyl ester-staining for each population shown in left. Mean fluorescence intensity (MFI) of cells shown in right (n=3 donors). **(G)** The 24 hours carboxyfluorescein diacetate succinimidyl ester-staining for each population shown in left. Cells MFI shown in right (n=3 donors). **(H)** The proportion of memory T cell (CD62L+, CD45RO+) in CD3 positive T cells measured by flow cytometry analysis (left) (n=3 donors). Representative flow cytometry analysis profiles of memory T cell in CD3 positive T cells in an independent donor out of 3 donors (right). **(A–H)** Values were expressed as the means ± SD. Unpaired t test was performed. ****p<0.0001, ***p<0.001, **p<0.01, *p<0.05 compared to RNAU6 anti-CD19 CAR-T cells.

To assess the influence of miR155 on the anti-tumor efficacy of anti-CD19 CAR-T cells, we co-cultured miR155 over-expressing anti-CD19 CAR-T cells with CD19-positive target cells. The results showed that miR155 expression significantly enhanced the cytolytic activity of anti-CD19 CAR-T cells compared with RNAU6 anti-CD19 CAR-T cells (**Figures 2A–C**). To assess the effects of miR155 on the stress activity of anti-CD19 CAR-T cells, three rounds of repeated antigen simulation and cytotoxicity assay were performed. The results indicated that anti-CD19 CAR-T cells might be gradually depleted under long-term antigen stimulation, while the cytotoxicity of anti-CD19 CAR-T cells was increased by miR155 and miR155 over-expression might be beneficial for the long-term anti-tumor function of anti-CD19 CAR-T cells (**Figure 2D**).

We further found that miR155 over-expressing anti-CD19 CAR-T cells showed increased secretion of TNF-α, an inflammatory cytokine that exerts cytotoxic effects on a wide range of tumor cells (24), compared with RNAU6 anti-CD19 CAR-T cells (**Figure 2E**). The miR155 over-expressing anti-CD19 CAR-T cells also showed increased secretion of IFN-γ, an antiviral cytokine that activates monocyte cytotoxicity (25), and increased secretion of IL-2, a critical cytokine for T cell activation and proliferation (26) (**Figure 2E**).

We next evaluated cell proliferation using the carboxyfluorescein diacetate succinimidyl ester method. At 0 hour, there was no significantly difference in carboxyfluorescein diacetate succinimidyl ester mean fluorescent intensity between the groups (**Figure 2F**). After 24 hours, significantly increased proliferation was observed in the miR155 over-expressing anti-CD19 CAR-T cells (**Figure 2G**). In addition, miR155 over-expressing anti-CD19 CAR-T cells showed more rapid expansion at day 19, and the survival rate showed improvement as well (**Figure S1I**).

A previous study reported that engineering less differentiated naive and/or memory T cells provides CAR-T cells with superior persistence *in vivo*, and promoting memory T cell formation has recently been used as a strategy to enhance CAR-T cell sustained persistence (27). We assessed the number of T_CM_ cells and confirmed a higher proportion of T_CM_ cells in miR155 over-expressing anti-CD19 CAR-T cells (**Figure 2H**).

Together, these results suggested that miR155 upregulation improved the function of CD19-specific CAR-T cells *in vitro*.

### LSD1 Downregulation Enhances the Function of CD19-Specific CAR-T Cells *In Vitro*

**Figure 3:**
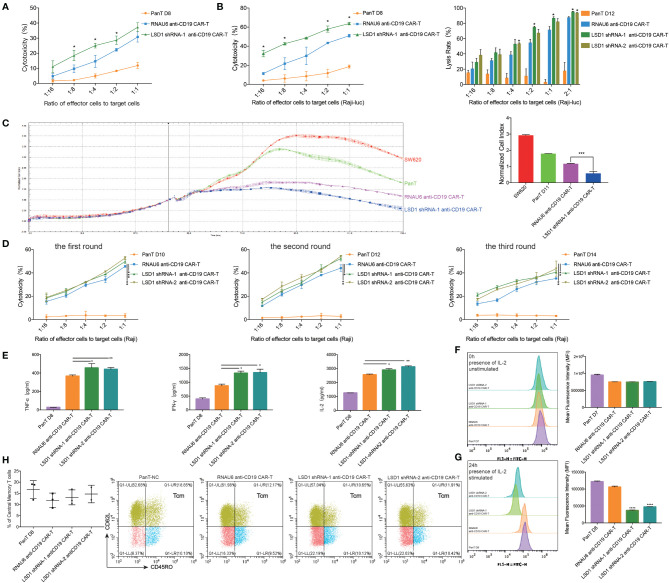
LSD1 downregulation enhanced the function of anti-CD19 CAR-T cells *in vitro*. **(A)** Apoptosis assay. LSD1 shRNA co-expressed anti-CD19 CAR-T cells were co-cultured with CD19-expressing Raji cells at a gradient E/T ratio for 12 hours. The apoptosis signals (Annexin V) of target cells were measured by flow cytometry analysis (n=3 donors). **(B)** The antitumor activity of LSD1 shRNA co-expressed anti-CD19 CAR-T cells against Raji-luc cells detected by apoptosis assay (left) and luciferase assay (right) (n=3 donors). **(C)** The antitumor activity of LSD1 shRNA co-expressed anti-CD19 CAR-T cells against hCD19-SW620 cells. The RTCA system was used to monitor CAR-T cells and hCD19-SW620 cells at an E/T ratio of 1:4 in the E-plates with impedance plotted over time (left). The quantitation of normalized cell index at the end of the assay was presented (right) (n=3 donors). **(D)** The stress test with repetitive antigen stimulation *in vitro*. LSD1 shRNA co-expressed anti-CD19 CAR-T cells and Raji cells were co-cultured at an E/T ratio of 1:1 for 48 hours with no CD19+ cells detected. After 48 hours, the CAR-T cells were then moved to a fresh set of Raji cells at a 1:1 E/T ratio. After each round, the apoptosis assay was proceeded, and 3 rounds of transfer over 6 days were performed (n=3 donors). **(E)** The secretory level of TNF-α (left), IFN-γ (middle) and IL-2 (right) of CAR-T cells after the co-culture with Raji cells at an E/T ratio of 1:1 for 12 hours, detected by ELISA (n=3 donors). **(F)** The proliferation of LSD1 shRNA co-expressed anti-CD19 CAR-T cells measured by carboxyfluorescein diacetate succinimidyl ester method. The stimulation referred to co-culturing with Raji cells at an E/T ratio of 1:2. The assay included continuous presence of IL-2. The 0 hour carboxyfluorescein diacetate succinimidyl ester-staining for each population shown in left. Mean fluorescence intensity (MFI) of cells shown in right (n=3 donors). **(G)** The 24 hours carboxyfluorescein diacetate succinimidyl ester-staining for each population shown in left. Cells MFI shown in right (n=3 donors). **(H)** The proportion of memory T cell (CD62L+, CD45RO+) in CD3 positive T cells measured by flow cytometry analysis (left) (n=3 donors). Representative flow cytometry analysis profiles of memory T cell in CD3 positive T cells in an independent donor out of 3 donors (right). **(A–H)** Values were expressed as the means ± SD. Unpaired t test was performed between the groups. ****p<0.0001, ***p<0.001, **p<0.01, *p<0.05 compared to RNAU6 anti-CD19 CAR-T cells.

To assess the influence of LSD1 on the anti-tumor efficacy of anti-CD19 CAR-T cells, we co-cultured LSD1 shRNA-expressing anti-CD19 CAR-T cells with CD19-overexpressing target cells. The results showed that the cytolytic activity of LSD1 shRNA-expressing anti-CD19 CAR-T cells was significantly increased compared with RNAU6 anti-CD19 CAR-T cells (**Figures 3A–C**). This increased anti-tumor cytolytic activity was further verified by the stress activity of anti-CD19 CAR-T cells in three rounds of the cytotoxicity assay. The cytotoxicity of anti-CD19 CAR-T cells was increased by expression of LSD1 shRNA (**Figure 3D**). These results suggested that LSD1 downregulation might be beneficial for the long-term anti-tumor function of anti-CD19 CAR-T cells.

The secretions of IFN-γ, TNF-α and IL-2 were enhanced in LSD1 shRNA-expressing anti-CD19 CAR-T cells (**Figure 3E**), and proliferation as evaluated by the carboxyfluorescein diacetate succinimidyl ester method was significantly increased (**Figures 3F, G**). Additionally, LSD1 shRNA-expressing anti-CD19 CAR-T cells showed better expansion and survival rate *in vitro* (**Figure S1J**), but no difference in the proportion of T_CM_ cells was observed (**Figure 3H**).

Together, our results showed that LSD1 downregulation enhanced the function of CD19-specific CAR-T cells *in vitro*.

### Anti-CD19 CAR-T Cells Efficiently Inhibit Tumor Progression *In Vivo*

**Figure 4:**
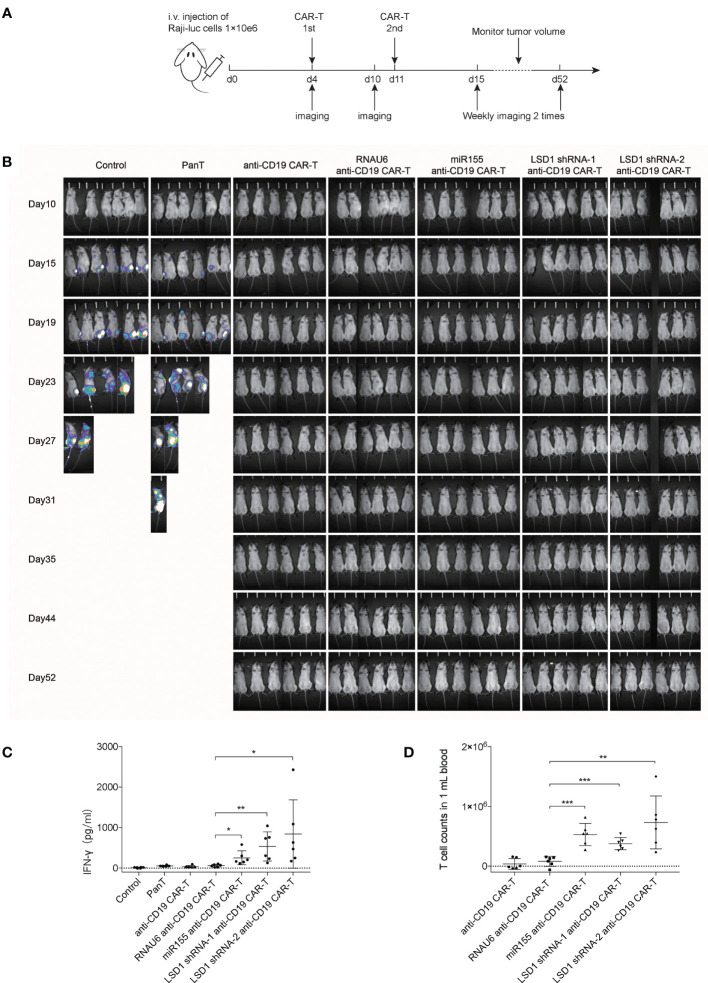
Anti-CD19 CAR-T cells efficiently inhibit Raji tumor progression *in vivo.* ***(*A*)*** Schema of study *in vivo* demonstrating the antitumor activity of CAR-T cells in a disseminated human B-cell malignancy xenogeneic NPG/Vst mice model (n=6). **(B)** Bioluminescent imaging of NPG mice *in vivo* weekly 2 times (n=6). **(C)** The levels of serum IFN-γ concentration in mice measured on the 7th day after the second CAR-T cells injection (n=6). **(D)** The numbers of T cells in blood of mice detected by flow cytometry analysis at day 52 when the study terminated (n=6). **(C, D)** Values were expressed as the means ± SD. Unpaired t test was performed. ***p<0.001, **p<0.01, *p<0.05 compared to RNAU6 anti-CD19 CAR-T cells.

To evaluate the anti-tumor activity of anti-CD19 CAR-T cells *in vivo*, we established a mouse xenograft model by injecting NPG mice with Raji-luc cells. After 4 days, the mice with established xenografts were randomly divided into seven groups; the mean total counts detected by *in vivo* bioluminescence imaging was 1.53 ± 0.3×10^4^ photons per sec (**Figure S2A**). CAR-T cells were injected with a dose of 1×10^8^/kg CAR-T cells on day 4 and 11 (**Figure 4A**). Tumor growth was monitored twice a week by *in vivo* bioluminescence imaging. The results revealed that miR155 expression or LSD1 shRNA expression in anti-CD19 CAR-T cells resulted in potent anti-tumor activity and complete tumor clearance *in vivo* (**Figures 4B** and **S2B**). The serum IFN-γ level in both miR155- and LSD1 shRNA-expressing anti-CD19 CAR-T cell groups, measured on the 7th day after the second CAR-T cell injection, were significantly increased (**Figure 4C**). Substantially increased numbers of T cells in the blood of miR155- and LSD1 shRNA co-expressing anti-CD19 CAR-T groups were detected at study termination compared to the early times (**Figures 4D** and **S2C, D**). The increased serum IFN-γ concentration and T cells proliferation indicated that the expression of miR155 and LSD1 shRNA had improved the antitumor activity and the proliferation of anti-CD19 CAR-T cells *in vivo* to some extent. Although there was no difference in survival rates and weight changes among the CAR-T cell groups (**Figures S2E, F**), which may be involved in xenograft complicate model and the difficulty to evaluate CAR-T cell efficacy *in vivo* using different dose of CAR-T cells. Nevertheless, these results indicated that the expression of miR155 and LSD1 shRNA improved the function of anti-CD19 CAR-T cells *in vivo*.

### Identification of miR155 Targets in Anti-CD19 CAR-T Cells

**Figure 5:**
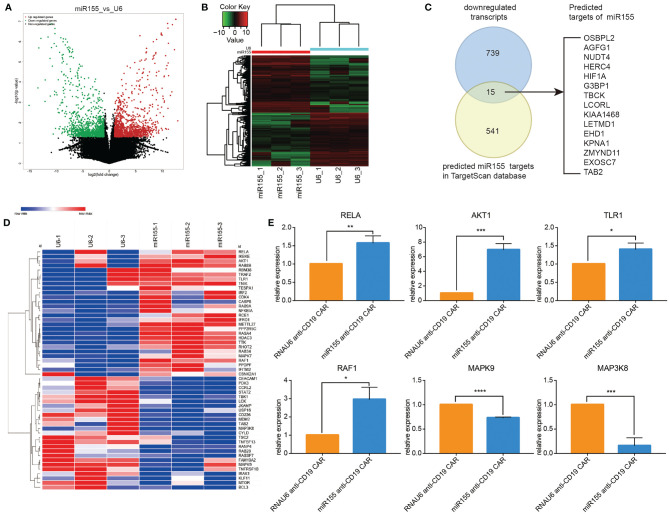
RNA sequencing revealed the miR155 targets in anti-CD19 CAR-T cells. **(A)** Volcano map of differential expression. **(B)** Cluster heat map of gene expression. **(C)** Predicted targets of miR155 in miR155 over-expressing anti-CD19 CAR-T cells. ﻿The Venn diagram showed the overlap between gene downregulated in miR155 over-expressing anti-CD19 CAR-T cells and predicted targets of miR155 in TargetScan dataset. **(D)** The cluster heat map of differential gene expression. **(E)** The verification of differential expression by RT-qPCR. Values were expressed as the means ± SD. Unpaired t test was performed. ***p<0.001, **p<0.01, *p<0.05 compared to RNAU6 anti-CD19 CAR-T cells.

Based on the positive role of miR155 on the anti-tumor function of anti-CD19 CAR-T cells, we next explored the putative target genes regulated by miR155 in miR155 over-expressing anti-CD19 CAR-T cells by RNA-seq. The volcano diagram and cluster analysis heat map results were shown in **Figures 5A, B**. A total of 1688 transcripts showed significant differential expression in miR155 over-expressing anti-CD19 CAR-T cells (*p* < 0.05). Among the 754 transcripts that were significantly downregulated in miR155 over-expressing anti-CD19 CAR-T cells, 15 transcripts were identified as ﻿predicted targets of miR155 by TargetScan (**Figure 5C**).

All differentially expressed transcripts were mapped to terms in the GO database (**Figure S3A**); the top 30 significant enriched GO terms were shown in **Figure S3B**. KEGG pathway analysis was performed in **Figure S3C** and the top 30 significant enriched pathways were shown in **Figure S3D**. The results indicated that GO terms related to immune system process and cell cycle process, and KEGG pathways related to the immune system and cell cycle were regulated by miR155.

Based on the results of GO terms, KEGG pathway and the functional annotations of genes, we selected genes of interest related to cell proliferation, growth, apoptosis inhibition and immune regulation processes to create a cluster heat map analysis (**Figure 5D**). To verify the reliability of the RNA-seq results, six differentially expressed genes (*RELA*, *AKT1*, *TLR1*, *RAF1*, *MAPK9*, *MAP3K8*) were randomly selected and examined in miR155 over-expressing anti-CD19 CAR-T cells by RT-qPCR. The results were consistent with the RNA-seq findings (**Figure 5E**).

### Identification of LSD1 Targets in Anti-CD19 CAR-T Cells

**Figure 6:**
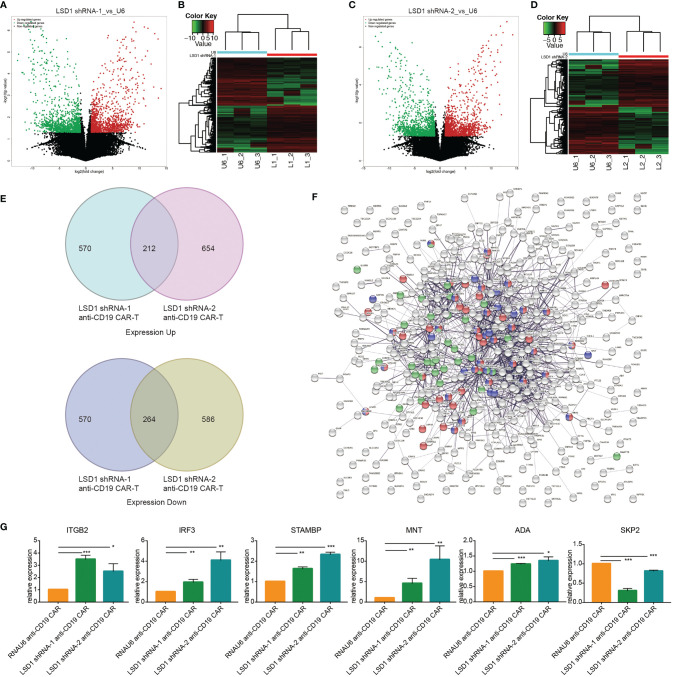
RNA sequencing revealed the target genes regulated by LSD1 in anti-CD19 CAR-T cells. **(A)** Volcano map of differential expression between LSD1 shRNA-1 co-expressing anti-CD19 CAR-T cells and RNAU6 anti-CD19 CAR-T cells. **(B)** Cluster heat map of gene expression in LSD1 shRNA-1 co-expressing anti-CD19 CAR-T cells and RNAU6 anti-CD19 CAR-T cells. **(C)** Volcano map of differential expression between LSD1 shRNA-2 co-expressing anti-CD19 CAR-T cells and RNAU6 anti-CD19 CAR-T cells. **(D)** Cluster heat map of gene expression in LSD1 shRNA-2 co-expressing anti-CD19 CAR-T cells and RNAU6 anti-CD19 CAR-T cells. **(E)** ﻿The Venn diagram shown the overlap between gene upregulated or downregulated in LSD1 shRNA-1 anti-CD19 CAR-T cells and LSD1 shRNA-2 anti-CD19 CAR-T cells. **(F)** ﻿Network of protein interaction analysis with the 212 up-expressing genes and 264 down-expressing genes in **(E)**. Red-colored ones associated with the term of cell cycle, purple-colored ones associated with the term of regulation of cell cycle, and green-colored ones associated with the term of immune effector process. **(G)** The verification of differential expression by RT-qPCR. Values were expressed as the means ± SD. Unpaired t test was performed. ***p<0.001, **p<0.01, *p<0.05 compared to RNAU6 anti-CD19 CAR-T cells.

Based on the positive role of LSD1 downregulation on the anti-tumor activity of anti-CD19 CAR-T cells, we investigated the putative target genes regulated by LSD1 in LSD1-expressing anti-CD19 CAR-T cells by RNA-seq. The volcano diagram and cluster analysis heat map results are shown in **Figures 6A–D**.

The differentially expressed genes in LSD1 shRNA-1 anti-CD19 CAR-T cells and LSD1 shRNA-2 anti-CD19 CAR-T cells were analyzed to obtain a set of transcripts with the same patterns of expression. Pairwise comparisons identified 212 transcripts that were simultaneously up-regulated in both cell groups and 264 transcripts that were simultaneously down-regulated in both cell groups (*p* < 0.05) (**Figure 6E**). We constructed a protein interaction network and performed enrichment analysis to determine the function of these differentially expressed transcripts. The differentially expressed transcripts were involved in cell cycle, regulation of cell cycle and immune effector process (**Figure 6F**). Among these terms, 6 differentially expressed genes (*ITGB2*, *IRF3*, *STAMBP*, *MNT*, *ADA*, *SKP2*) were randomly selected and tested by RT-qPCR to verify the reliability of the results of RNA-seq. The results were consistent with the results of RNA-seq (**Figure 6G**).

## Discussion

The co-expression of miRNA or shRNA in anti-CD19 CAR-T cells may enhance anti-tumor function. The first CAR-T cell therapy was approved by the FDA in 2017, and subsequent research has indicated the requirements for further enhancements of the anti-tumor efficacy and persistence of CAR-T cells to improve the prognosis of cancer patients (28). Engineering T cells with a partial increase of effector-associated factors and inhibition of exhaustion-associated factors may be promising strategies for cancer immunotherapies (29). The use of combination therapy with checkpoint receptor-blocking antibodies and CAR-T cell therapy in cancer treatment has been demonstrated to be safe and effective (30). Our approach of integrating miRNA or shRNA cassettes into CAR-expressing retroviral vectors reflects potentially significant opportunities for the improvement of currently established protocols for CAR engineering strategies.

We have successfully constructed a novel cell therapy by co-expressing miRNA or shRNA with anti-CD19 CAR in human T cells, with an U6 promoter driving the expression of miR155 or LSD1 shRNA and an EF1α promoter driving the expression of anti-CD19 CAR. High titer of vectors were successfully produced; miR155 or LSD1 shRNA anti-CD19 CAR T cells were successfully manufactured with efficient anti-CD19 CAR and miR155 or LSD1 shRNA expression, leading to synchronous regulation of CAR-T cells. The simultaneous expression of miR155 or LSD1 shRNA did not affect the expression of anti-CD19 CAR. This design provides a promising therapeutic approach for the optimization of anti-CD19 CAR-T cells and identification of the novel regulators using shRNA-based screen.

MiR155 overexpression enhanced the function of CD19-specific CAR-T cells. The enhanced cytolytic activity and the increased serum IFN-γ and TNF-α concentration *in vitro*, as well as the increased serum IFN-γ concentration *in vivo* indicated that miR155 co-expression improved the anti-tumor function of anti-CD19 CAR-T cells. The stress test, performed after three rounds of repeated antigen challenge, indicated that the conventional anti-CD19 CAR-T cells might be gradually exhausted under long-term antigen challenge, while the cytotoxicity of anti-CD19 CAR-T cells was increased upon co-expression of miR155, suggesting that miR155 over-expression might be beneficial for the long-term anti-tumor function of anti-CD19 CAR-T cells. Besides, the killing advantage of miR155 co-expressing anti-CD19 CAR-T cells to hCD19-SW620 cells showed its potential of application on solid tumors, not just on blood tumors.

As to the aspect of proliferation function, miR155 over-expressing anti-CD19 CAR-T cells showed significantly better expansion and survival rate *in vitro* and increased proliferation *in vitro* and *in vivo*. It has been demonstrated that poor persistence of infused CAR-T cells can limit an effective and long-term antitumor immune response *in vivo* (31). The increased CAR-T cells proliferation is beneficial for the improvement of the outcome of long-term therapy. The application of miR155 overexpression in CAR-T cell therapy may overcome the limitations of CAR-T cell senescence and functional exhaustion.

Studies showed that miRNAs regulate T cell differentiation and function (32). miR155 was one of the first miRNAs shown to be induced during the inflammatory response. miR155 was regarded as an inflamma-miR because of its strong association with immune-related factors, including TNF-α, toll-like receptors and NF-κB (33). Researches have shown that miR155 upregulation promotes the differentiation of naive CD4+ T cells into Th1 cells through the regulation of the IFN-γ signal (34), which is of great significance for T cells to exert anti-tumor function for the secretion of IFN-γ, TNF-α, IL-2 and other cytokines. IFN-γ and IL-2 promote the proliferation and differentiation of cytotoxic T lymphocytes and enhance the anti-tumor immune response, while TNF-α induces the apoptosis of target cells and contributes to the anti-tumor function. Besides, miR155 enhances CD8+ T cell anti-tumor efficacy and proliferative activity by inhibiting T cell terminal differentiation and functional exhaustion through downregulation of the Akt inhibitor Ship1 and promoting expression of the polycomb repressor complex 2 (PRC2)-associated factor Phf19, indirectly leading to increased PRC2 activity (35). In addition, miR155 overexpression promotes T cell activation and proliferation by decreasing the expression of cytotoxic T lymphocyte-associated antigen 4 (36) and inhibits T cell apoptosis by inhibiting the BIM activation-related transcription factor FOXO3 (37).

Besides, it is increasingly clear that less differentiated naive and memory T cells are superior to effector T cells in CAR-T cell therapy, and one of the recent strategies to improve CAR-T cell persistence is focusing on memory cell formation (38). In this study, increased level of T_CM_ cells in miR155 co-expressing anti-CD19 CAR T cells might be associated with the long-term T cell persistence, consistent with the discovery that miR155 regulated the transcription factor T-bet to promote the differentiation of CD8+ T cells into memory T cells by targeting to SHIP-1, thus improving the proliferation and long-term persistence of CD8+ T cells (37, 39, 40).

RNA-seq analysis showed that miR155 targets may be involved in pathways related to immune response and cell cycle. The up-regulation of *RELA*, *AKT1*, *TLR1* and *RAF1* and the down-regulation of *MAPK9* and *MAP3K8* were determined by RT-qPCR, indicating that the enhanced anti-tumor function may associate with T cell immune response and proliferation related Toll-like receptor-Akt-NF-κB signaling pathway. And targets of miR155 may be involved in up-regulation of T cell receptor signaling pathway, immune response-regulating signaling pathway, negative regulation of apoptotic process, cell proliferation, inflammatory response, cytokine production and cell activation.

LSD1 shRNA overexpression enhanced the function of CD19-specific CAR-T cells. The enhanced cytolytic activity and the increased serum IFN-γ and TNF-α concentration *in vitro*, as well as the increased serum IFN-γ concentration *in vivo* indicated that LSD1 shRNA co-expression improved the anti-tumor function of anti-CD19 CAR-T cells. The stress test indicated that LSD1 down-expression might be beneficial for the long-term anti-tumor function of anti-CD19 CAR-T cells. And LSD1 down-expressing anti-CD19 CAR-T cells showed significantly better expansion and survival rate *in vitro* and increased proliferation *in vitro* and *in vivo*.

LSD1 is involved in the regulation of a variety of biological processes, and first discovered as part of the C-terminal binding protein-1 co-repressor complex (41). LSD1 plays a critical role in the formation of transcriptional repressor complexes with histone deacetylase 1 and 2 and the RE1 silencing transcription factor co-repressor (42) and regulates gene activation and repression (43). Recent studies demonstrated that LSD1 inhibition is correlated with potent anti-tumor T cell immunity and enhanced T cell infiltration in the tumor microenvironment in mouse models (44). LSD1 plays an important role in the regulation of Th1 cell differentiation, and the inhibition of LSD1 in activated CD4+ T cells induced IFN-γ-producing Th1 cells (45), suggesting that LSD1 inhibition may be a potential strategy to improve Th1 cell differentiation and cytotoxic T cell immune function. Other studies reported that LSD1 is a negative regulator of the response to inflammation in hematopoietic stem cells during the endotoxic shock that is typically observed during acute bacterial or viral infection (22). In addition, LSD1 inhibition promoted the expansion of hematopoietic stem cells derived from human umbilical cord blood and increased the number of transplantable hematopoietic stem cells (46), suggesting that LSD1 inhibition might exert a regulatory effect on cell differentiation and cell proliferation.

RNA-seq analysis indicated that LSD1 may be involved in pathways altered related to immune response, cell cycle and the regulation of cell cycle. Among these differentially expressed genes, the increased expression of *ITGB2*, *IRF3*, *STAMBP*, *MNT* and *ADA* and the decreased expression of *SKP2* were determined by RT-qPCR, indicating that LSD1 may be involved in the up-regulation of cell proliferation, T cell activation, T cell receptor signaling pathway, immune response, I-κB kinase/NF-κB signaling and cytokine secretion, and the down-regulation of apoptotic signaling. However, the detailed mechanisms underlying these effects remain to be elucidated in the future.

Our study provides a novel strategy for cancer treatment involving the integration of miRNA or shRNA into CAR-T cells. This approach can be used to enhance the function of CAR-T cells and may represent a new preclinical model in cancer immunotherapy research. The functional advantage provided by co-expressing miR155 or LSD1 shRNA were demonstrated preliminarily *in vitro* and *in vivo*. However, more extensive and longer trials are needed to explore the dose and time of CAR-T cells injection to make the difference in tumor imaging between groups. Besides, the mechanism and side effects of this application have not yet been fully verified. More studies are required to develop and optimize these CAR-T cells to maximize their clinical activity.

## Data Availability Statement

The data presented in the study are deposited in the NCBI Trace Archive NCBI Sequence Read Archive repository, accession number PRJNA773988.

## Ethics Statement

The studies involving human participants were reviewed and approved by the Biomedical Research Ethics Committee of the Beijing University of Chinese Medicine. The patients/participants provided their written informed consent to participate in this study. The animal study was reviewed and approved by the Biomedical Research Ethics Committee of the Beijing University of Chinese Medicine.

## Author Contributions

JW conceived the project and supervised the experiments. JZ and JJZ performed the experiments and analyzed the data with the help of XL, YF, SH, QJ, and BS. QW and DW provided critical reagents and protocols. GZ and ZC performed the bioinformatics analysis. JZ wrote the manuscript with revisions by JW. All authors approved the submitted version.

## Funding

This work was supported by the “Double First-Class” initiative to Beijing University of Chinese Medicine (Grant number 1000041510155).

## Conflict of Interest

The authors declare that the research was conducted in the absence of any commercial or financial relationships that could be construed as a potential conflict of interest.

## Publisher’s Note

All claims expressed in this article are solely those of the authors and do not necessarily represent those of their affiliated organizations, or those of the publisher, the editors and the reviewers. Any product that may be evaluated in this article, or claim that may be made by its manufacturer, is not guaranteed or endorsed by the publisher.
