# Supplementary material for: Co-Expression of miR155 or LSD1 shRNA Increases the Anti-Tumor Functions of CD19 CAR-T Cells
Source: Front Immunol. 2022 Jan 3;12:811364. doi: 10.3389/fimmu.2021.811364 (PMC8761951; doi:10.3389/fimmu.2021.811364)
Supplement: Supplementary file 1 [file DataSheet_1.docx]

**Supplemental Material**

**
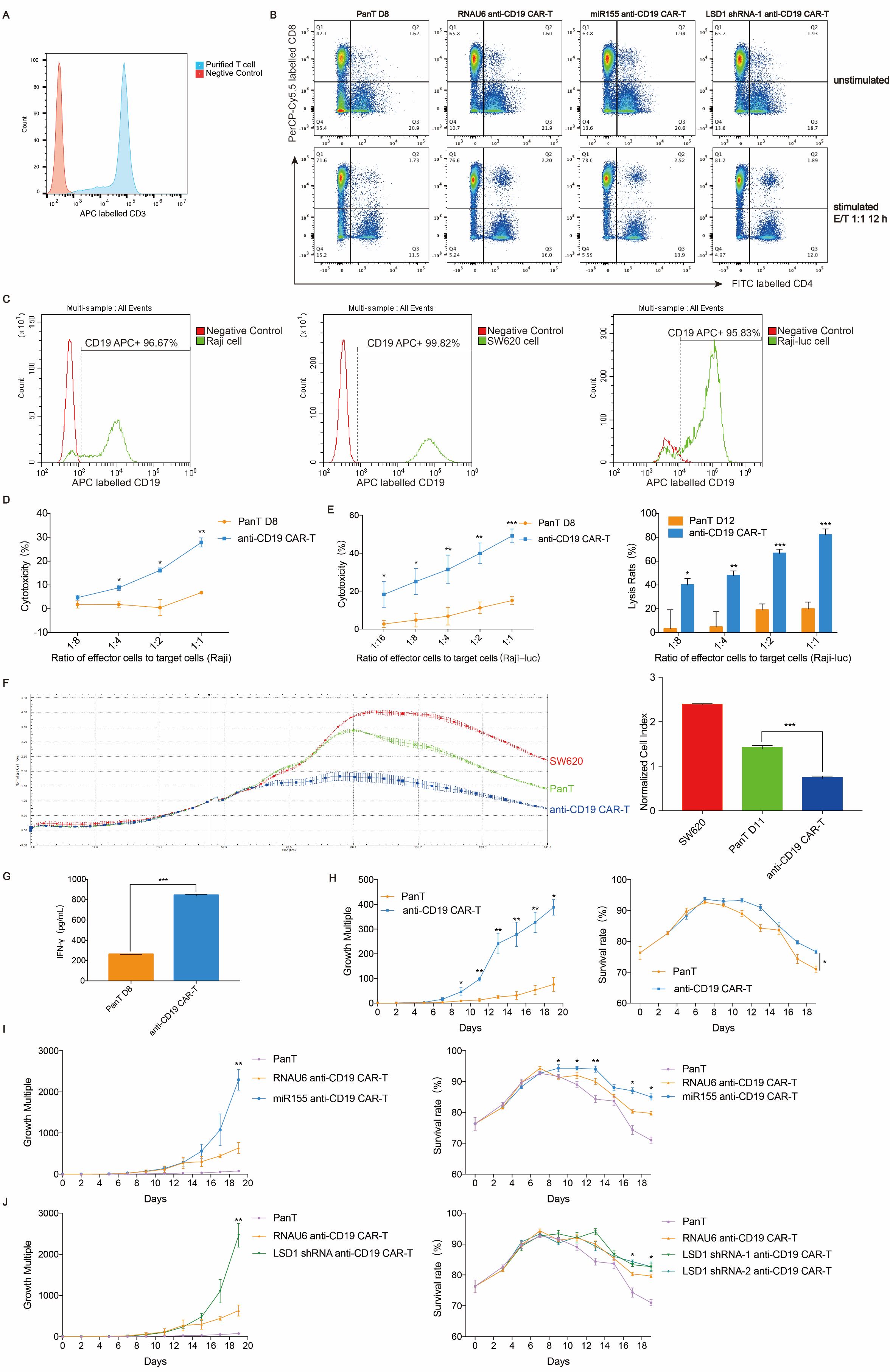
**

**Fig S1. Anti-CD19 CAR-T cells elicit antitumor reactivity against CD19-expressing target cells.**

(A) The purity of T cells after isolation and activation detected by FCA.

(B) The ratios of CD4+/CD8+ T cells in CD3+ T cells stimulated without Raji cells (up) or with Raji cells (down).

(C) The expression of CD19 in target cells Raji, SW620 and Raji-luc cells analyzed by FCA.

(D) The apoptosis assay of anti-CD19 CAR-T cells. Anti-CD19 CAR-T cells were co-cultured with CD19-expressing Raji cells at a gradient of E/T ratio for 12 hours. The apoptosis signals (Annexin V) of target cells were measured by FCA (n=3 donors).

(E) The antitumor activity of anti-CD19 CAR-T cells against Raji-luc cells detected by apoptosis assay(left) and luciferase assay(right) (n=3 donors).

(F) The antitumor activity of anti-CD19 CAR-T cells against CD19-expressing SW620 cells measured by RTCA. The RTCA system was used to monitor anti-CD19 CAR-T cells and SW620 cells at an E/T ratio of 1:4 in the E-plates with impedance plotted over time (left). The quantitation of normalized cell index at the end of the assay was presented (right) (n=3 donors).

(G) The level of released IFN-γ of anti-CD19 CAR-T cells after the co-culture with Raji cells for 12 hours, detected by ELISA. Error bars show standard error of the mean, triplicate determinations (n=3 donors).

(H) The growth multiple (left) and survival rate (right) of anti-CD19 CAR-T cells measured by cell counts every 48 hours (n=3 donors).

(I) The growth multiple (left) and survival rate (right) of miR155 co-expressing anti-CD19 CAR-T cells measured by cell counts every 48 hours (n=3 donors).

(J) The growth multiple (left) and survival rate (right) of LSD1 shRNA co-expressing anti-CD19 CAR-T cells measured by cell counts every 48 hours (n=3 donors).

(D-J) Values were expressed as the means ± SD. Unpaired t test was performed.

(D-H) ****p*＜0.001, ***p*＜0.01, **p*＜0.05 ﻿compared to Pan-T cells.

(I-J) ***p*＜0.01, **p*＜0.05 ﻿compared to RNAU6 anti-CD19 CAR-T cells.

**
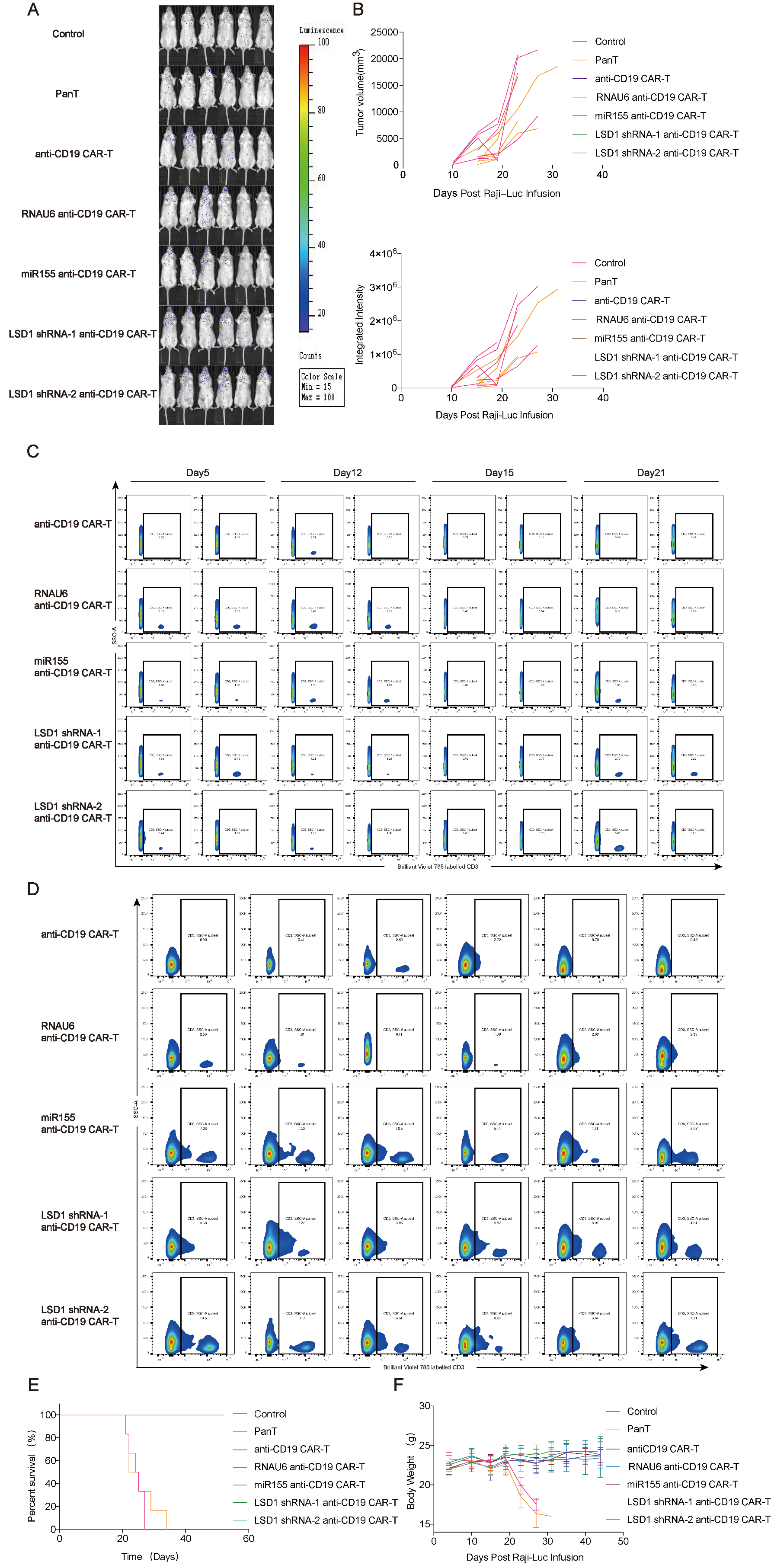
**

**S2. Anti-CD19 CAR-T cells efficiently inhibit Raji tumor progression *in vivo***

(A) Bioluminescent imaging *in vivo* of NPG mice with established xenografts at day 4 (n=6).

(B) Summary of the bioluminescence signal as a measurement of tumor growth after tumor cell infusion. The area of the tumor (up); the integrated intensity of tumor bioluminescence signal (down) (n=6).

(C) Representative FCA profiles of the percentage of T cell number in blood detected by FCA at day 5, day 12, day 15 and day 21 after the infusion of tumor cells (n=2).

(D) Representative FCA profiles of the percentage of T cell number in blood detected by FCA at day 52 when the study terminated (n=6).

(E) Survival rates of xenograft mice receiving PanT and CAR-T cells treatment (n=6).

(F) Body weight of xenograft mice receiving PanT and CAR-T cells treatment (n=6).

**
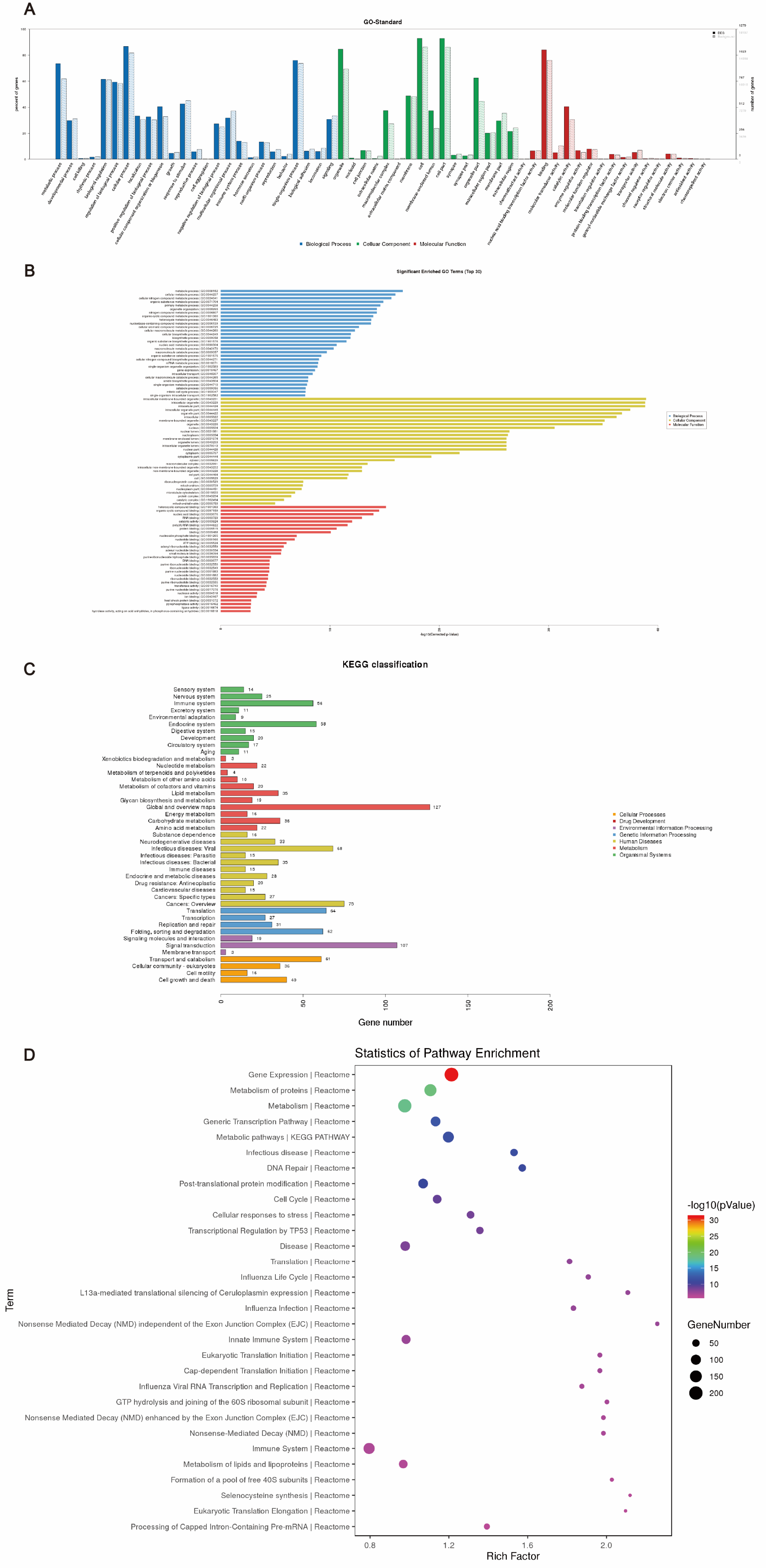
**

**S3. The enrichment analysis of RNA-seq in miR155 co-expressing** **anti-CD19 CAR-T cells compared with RNAU6 anti-CD19 CAR-T cells**

(A) GO analysis of differential expression.

(B) Top30 terms of GO analysis of differential expression.

(C) KEGG enrichment analysis.

(D) Top30 pathways of KEGG enrichment analysis.

**Supplementary Table 1 RT-qPCR primers**

| Gene | Primer | Sequence |
| --- | --- | --- |
| vector copy detection primer | jw003 | GACACCAGACTAAGAACCTAGAAC |
|  | jw004 | CTCAAAGTAGACGGCATCGCAGCT |
| *Human glyceraldehyde-3-phosphate dehydrogenase* (*GAPDH*) | jw403 | CATGTTCGTCATGGGTGTGAACCA |
|  | jw404 | ATGGCATGGACTGTGGTCATGAGT |

The sequences of primer for the vector copy and vector copy number integration per T cell detection.

**Supplementary Table 2 RT-qPCR primers**

| Gene | Primer | Sequence |
| --- | --- | --- |
| LSD1 | jw389 | ACATCTGCAGTCCAAAGGATGGGA |
|  | jw390 | GTACTGCCAACATGCCCGAACAAA |
| *human actin, beta* (*ACTB*) | jw401 | ACCAACTGGGACGACATGGAGAAA |
|  | jw402 | TAGCACAGCCTGGATAGCAACGTA |

The sequences of primer for LSD1 expression detection.
